# Methyl jasmonate promote protostane triterpenes accumulation by up-regulating the expression of squalene epoxidases in *Alisma orientale*

**DOI:** 10.1038/s41598-019-54629-6

**Published:** 2019-12-02

**Authors:** Rong Tian, Wei Gu, Yuchen Gu, Chao Geng, Fei Xu, Qinan Wu, Jianguo Chao, Wenda Xue, Chen Zhou, Fan Wang

**Affiliations:** School of Pharmacy, Nanjing University of Chinese Medicine, Nanjing, 210023 China

**Keywords:** Plant sciences, Molecular biology

## Abstract

Protostane triterpenes, which are found in *Alisma orientale*, are tetracyclic triterpenes with distinctive pharmacological activities. The natural distribution of protostane triterpenes is limited mainly to members of the botanical family Alismataceae. Squalene epoxidase (SE) is the key rate-limiting enzyme in triterpene biosynthesis. In this study, we report the characterization of two SEs from *A. orientale*. AoSE1 and AoSE2 were expressed as fusion proteins in *E. coli*, and the purified proteins were used in functional research. *In vitro* enzyme assays showed that AoSE1 and AoSE2 catalyze the formation of oxidosqualene from squalene. Immunoassays revealed that the tubers contain the highest levels of AoSE1 and AoSE2. After MeJA induction, which is the main elicitor of triterpene biosynthesis, the contents of 2,3-oxidosqualene and alisol B 23-acetate increased by 1.96- and 2.53-fold, respectively. In addition, the expression of both AoSE proteins was significantly increased at four days after MeJA treatment. The contents of 2,3-oxidosqualene and alisol B 23-acetate were also positively correlated with *AoSEs* expression at different times after MeJA treatment. These results suggest that AoSE1 and AoSE2 are the key regulatory points in protostane triterpenes biosynthesis, and that MeJA regulates the biosynthesis of these compounds by increasing the expression of *AoSE1* and *AoSE2*.

## Introduction

*Alisma orientale* is one of the most important perennial medicinal plants in traditional Chinese medicine, where its rhizomes have been used for nearly 2000 years to eliminate “dampness”, reduce edema, and promote urinary excretion^1^. Protostane triterpenes from * A. orientale* have unique structural features that include a β-CH_3_ on C_10_ and C_14_, an α-CH_3_ on C_8_, and an *S*-configuration on C_20_^2^. The protostane triterpenes are a group of rare natural plant compounds that are derived from alisol B 23-acetate, which is a natural product present in the rhizomes of *A. orientale*. Protostane triterpenes have a wide range of pharmacological activities and can be used to treat HIV, HBV, cancer, hyperlipidemia, atherosclerosis, allergies, inflammation, fatty liver, and hyperglycemia^3–9^. Alisol B 23-acetate is the main active component, and is used as the general evaluation indexcha for quality in *A. orientale*^10^. Recent studies have shown that alisol B 23-acetate from *A. orientale* can restore the sensitivity of multi-drug resistant cells to anti-tumor drugs, because it may take part in the transport of P-glycoprotein^11^. However, these compounds are found only in a few plant groups such as *Alisma*, and in very low amounts. In addition, the unique structure of protostane triterpenes makes them very difficult to synthesize through chemical synthesis. The further development and utilization of these compounds is thus limited by these traits.

Synthetic biology is an effective way to address the scarcity of resource components^12,13^, and the yield of the active component can be increased by increasing the efficiency of biosynthesis in the plant^14,15^. Triterpenes are synthesized by the mevalonate (MVA) pathway in the cytoplasm^16^. Based on published research and our preliminary findings, the possible steps in protostane triterpenes biosynthesis that need to be optimized are shown in Fig. S1. HMGR (3-hydroxy-3-methylglutaryl-CoA reductase), SS (squalene synthetase), FPPS (farnesyl pyrophosphate synthase), SE (squalene epoxidase), and OSC (oxidosqualene cyclases) are thought to be key enzymes in triterpenes  biosynthesis. HMGR catalyzes the formation of MVA from 3-hydroxy-3-methylglutary CoA (HMG-CoA) and a triphosphopyridine nucleotide (NADPH)^17^, and MVA is then converted to isopentenyl pyrophosphate (IPP). Farnesyl pyrophosphate (FPP) is produced from the condensation of IPP and dimethylallyl pyrophosphate (DMAPP) by the action of FPPS. SS catalyses the conversion of two molecules of FPP to form squalene. An oxygen atom is then inserted into the C = C bond of squalene by SE to convert it to oxidosqualene, and the triterpene skeleton is formed by OSC. Finally, alisol B 23-acetate is formed via functional group modification and is then transformed into various *Alisma* protostane triterpenes, of which the formation of the protostane triterpene skeleton is the core biosynthetic step.

SE catalyzes the conversion of squalene to 2,3-oxidosqualene, which is the precursor of the triterpene skeleton. This enzyme is a non-cytochrome P450-type monooxygenase that participates in triterpene biosynthesis and functions as a rate-limiting step in the pathway^18^. At present, *SE* genes have been cloned from pharmacological plants such as *Panax ginseng*, *Panax notoginseng*, *Acanthopanax senticosus*, and *Ginkgo biloba*^19–22^. The expression of *GbSE* in roots can promote the biosynthesis of triterpenoid saponins in *G. biloba*^22^. Up-regulation of *PgSE* expression causes the accumulation of ginsenosides in *P. ginseng*^21^. These results tend to confirm that SE is a key enzyme involved in the synthesis of terpenes. In previous studies on the key enzymes in the protostane triterpenes biosynthetic pathway in *A. orientale*, we cloned and characterized *AoHMGR*, *AoFPPS*, and *AoSS* (GenBank accession numbers KP342318, HQ724508, and JX866770, respectively)^23–25^, while characterization and functional analysis of SE in *A. orientale* has not yet been reported.

In 1971, jasmonic acid (JA) was first isolated as a plant growth hormone^26^. Jasmonates [JA, methyl jasmonate (MeJA), and related compounds] are lipid-derived signal molecules that have been shown to play important roles in the regulation of plant growth and development^27,28^. MeJA can regulate metabolic pathways and reaction rates through a series of signal transduction processes in the cells^29^. MeJA acts through a receptor in the plant cell membrane to regulate the expression of the key enzyme genes and transcription factors in biosynthetic pathways, and it can promote the production of secondary metabolites in plants^30^. The effect of MeJA on triterpene saponin biosynthesis has been reported in *P. ginseng*. The expression of *PgSE* and the ginsenoside content are both increased in ginseng hairy or adventitious root cultures after MeJA treatment. The expression levels of *AvHMGR* and *AvDXR*, which are the key enzyme genes for terpene synthesis in *Amomum villosum*, were increased significantly in response to MeJA induction^31^. Although these studies provide some insight into MeJA function with respect to gene expression and metabolite content, there is a need to examine the differences in gene expression and protein and metabolite contents, since mRNA levels are not always consistent with protein levels due to post-transcriptional, translational, and post-translational regulation.

Therefore, we first cloned *AoSE* genes and then performed prokaryotic expression to identify the function of the AoSE proteins. We then prepared polyclonal antibodies to the AoSEs and determined their expression levels using immunodetection. We also analyzed the levels of the AoSE proteins and the alisol B 23-acetate contents at different growth stages in *A. orientale*. Finally, in order to evaluate the effects of exogenous MeJA on the levels of the AoSE proteins and the accumulation of 2,3-oxidosqualene and alisol B 23-acetate, we performed an induction experiment and analyzed the correlation between the AoSE levels and the alisol B 23-acetate content in *A. orientale*. The results of our study will provide important information on the triterpene biosynthetic pathway in *A. orientale*, and will also establish a foundation for regulating the production of large amounts if these bioactive components by MeJA treatment.

## Materials and Methods

### Plant materials and MeJA treatment

Plant materials were collected from Jianou in Fujian Province and identified as *Alisma orientale* by Professor Gu Wei (College of Pharmacy, Nanjing University of Chinese Medicine). Beginning on October 15th, leaves, tubers, and roots of *A. orientale* were collected every 15 days.

Seedlings of *A*. *orientale* were divided into the control and sample groups. MeJA dissolved in distilled water was applied to the leaves at a final concentration of 300 μM. Leaves of the control group were treated with an equal volume of distilled water. The water control and MeJA solution were sprayed until the leaf surfaces were saturated. All plants were sampled at 0, 1, 2, 3, 4, and 5 days after treatment. Plants were rinsed with distilled water and dried using tissue paper. Subsequently, plant biomass (fresh weight) was determined for 10 complete plants in each group (each plant was an individual sample). All treatments were performed in five replicates.

One-half of each sample was frozen in liquid nitrogen and stored at −80 °C to be used for RNA and protein extraction, and the other half was oven-dried at 60 °C to a constant weight for extraction and HPLC analysis. The dry samples (0.5 g) were extracted with 20 ml of acetonitrile in an ultrasonic bath for 30 min, filtered through a 0.45 µm membrane, and assayed by HPLC.

### HPLC analysis

Samples were analyzed using a Waters 2695 series HPLC system (Waters Corporation, Milford, MA USA), equipped with a quaternary pump and a variable wavelength ultraviolet (UV) detector. The samples (20 μL) were applied to a C_18_ analytical column (5 μm, 4.6 × 250 mm; Phenomnex, Torrance, CA, USA) at a flow rate of 1 mL/min. The mobile phase consisted of acetonitrile (A) and distilled water (B) and the gradient of the mobile phase was as follows: 0–10 min, 30% to 50% solvent A; 10–50 min, 50% to 90% solvent A. The column temperature was maintained at 25 °C, and the detection wavelength was set at 210 nm. All other reagents were of analytical grade. The contents of 2,3-oxidosqualene and alisol B 23-acetate were examined simultaneously. The alisol B 23-acetate standard (purity >98%) was provided by Prof. Peng Guo-Ping (Nanjing University of Traditional Chinese Medicine, Nanjing, China), and 2,3-oxidosqualene (purity >92%) was purchased from Sigma-Aldrich Company (St. Louis, MO USA).

### Cloning the full-length *AoSE1* and *AoSE2* cDNAs

#### RNA isolation

Total RNA was extracted from *A. orientale* roots, tubers, and leaves using the RNAsimple Total RNA kit (Tiangen, catalog number DP419). RNA samples were subjected to electrophoresis on 1.0% agarose gels and quantified by electronic digital imaging using the Syngene G:Box. RNA yield and purity were assessed using an Eppendorf UV-Vis spectrophotometer.

#### cDNA synthesis

Total RNA (2 µg) was reverse transcribed into cDNA using an oligo (dT)_18_ primer and the RNA PCR kit (AMV) version 3.0 (TaKaRa). The reactions were performed using the following temperature profile: 30 °C for 10 min, 42 °C for 30 min, 99 °C for 5 min, and 5 °C for 5 min.

Using cDNA synthesized from *A*. *orientale* leaf RNA as the template, conserved fragments of the *SE* gene were amplified and cloned using degenerate primers (AF and AR; Table S1), which were synthesized by Sangon Biotech Company (Shanghai, China).

#### Rapid amplification of cDNA ends

Specific primers were designed based on the sequences of the conserved fragments of the *AoSE1* and *AoSE2* genes and synthesized by Sangon Biotech Company (Shanghai, China) (primer pairs SF1 and SR1, and SF2 and SR2; Table S1). The 5′- and 3′-ends of the cDNAs were amplified using the SMARTer^TM^ RACE cDNA amplification kit (Takara Bio Inc.). The PCR products were cloned and sequenced by Shanghai Sangon Biotech company. The 5′- and 3′-ends were combined based on the overlapping region of the conserved and amplified fragments.

#### DNA sequencing of the full-length SE cDNAs

Primers for amplification of the full-length *SE* cDNAs were designed based on the combined sequences and synthesized by the Sangon Biotech Company (Shanghai, China) (primer pairs SF3 and SR3, and SF4 and SR4; Table S1). Each assay was conducted in a volume of 50 µL containing 2.0 µL template cDNA, 10 nmol/L of each primer, 5.0 µL 10 × PCR buffer, 0.2 mmol/L dNTPs, 1.5 mmol/L MgCl_2_, and 5 U rTaq DNA polymerase (TaKaRa). The temperature profile was as follows: a 3 min pre-denaturation at 95 °C, followed by 30 cycles of 95 °C for 30 s, 56 °C for 60 s, and 72 °C for 2 min. An additional 5 min extension at 72 °C was added to complete the amplification. PCR products were cloned and sequenced by the Sangon Biotech Company (Shanghai, China).

### Prokaryotic expression

#### Construction and identification of the prokaryotic expression vectors

Based on the *AoSE1* and *AoSE2* sequences obtained previously, primers were redesigned using sequences of the cloning vector pGHn-AoSEs (SF5/SR5 and SF6/SR6; Table S1). The reactions (50 µL) contained 5.0 µL 10 × PCR buffer, 4.0 µL 25 mmol/L MgCl_2_, 10 mmol/L dNTPs, 10 nmol/L of each primer, 5.0 µL cDNA template, 0.5 µL (5 U) rTaq enzyme, and 28.5 µL ddH2O. Amplification conditions were as follows: a 5 min denaturation step at 94 °C, followed by 30 cycles of 94 °C for 30 s, 54 °C for 30 s, and 72 °C for 1 min. An additional 1 min extension at 72 °C was added to complete the amplification. PCR products were examined by agarose gel electrophoresis, purified, cloned, and sequenced. The *AoSE1* gene was cloned into the pCzn1 expression vector between the KpnI and XbaI sites. The target gene was fused with the 6 × His-tag protein, resulting in the plasmid pCzn1-AoSE1. *AoSE2* was cloned into the pCzn1 expression vector between the KpnI and EcoRI sites. The target gene was fused with the 6 × His-tag protein, resulting in the plasmid pCzn1-AoSE2.

#### Expression of the recombinant proteins

Recombinant plasmids pCzn1-AoSE1 and pCzn1-AoSE2 (without mutations) were transformed into *E. coli* BL21 (Rosetta) cells. The positive clones were screened by PCR. Bacterial colonies were transferred into 3 mL LB containing 50 μg/mL ampicillin and cultured in 37 °C at 220 r·min^−1^ overnight. A 250 μL aliquot of each culture was then transferred to 30 mL LB medium containing 50 μg/mL ampicillin and cultured at 37 °C for about 2 h until the OD_600_ = 0.6–0.8. The cells were recovered by centrifugation at 10,000 × g at 25 °C for 2 min. The supernatants were removed, and the pellets were suspended in 100 μL buffer containing 0.2 mM IPTG and shaken at 220 r·min^−1^ for 12 h at 11 °C. After induction of protein expression, the cultures were centrifuged to pellet the bacterial cells. The supernatants from the induced cultures were then analyzed by 10% sodium dodecyl sulfate polyacrylamide gel electrophoresis (SDS-PAGE).

#### Expression and purification of the target proteins

The induced bacterial cultures were centrifuged at 10,000 r·min^−1^ for 20 min at 4 °C, and the pellets were suspended and mixed with 20 mL bacterial lysis buffer (20 mM Tris-HCl containing 1 mM phenylmethylsulfonyl fluoride [PMSF] at pH 8). The cell suspensions were lysed by ultrasonication for 20 min and centrifuged at 10,000 r·min^−1^ for 20 min at 4 °C. The pellets were washed three times using an inclusion body washing solution (20 mM Tris, 1 mM EDTA, 2 M urea, 1 M NaCl, 1% Triton X-100, pH 8) and lysis buffer (20 mM Tris, 5 mM DTT, 8 M urea, pH 8), and the precipitate was dissolved. The collected supernatants were stored for 12 h at 4 °C and then centrifuged at room temperature for 15 min. The solubilized inclusion bodies were added drop-wise to 20 mM Tris (pH = 8). Buffer was gradually added to achieve a 4-fold dilution, and the solution was slowly stirred until the urea concentration reached 0.5 M. The protein solution was placed in a dialysis bag and immersed overnight at 4 °C in 20 mM phosphate-buffered saline (PBS) at pH 7.4. The target proteins were then analyzed by 10% SDS-PAGE.

### *In vitro* characterization of the activities of AoSE1 and AoSE2

Reaction mixtures (500 µL) contained 0.1 mM DTT, 25 mM MgCl_2_, 490 µM squalene, 0.14 mM NADPH, 0.5% Triton X-100, 1 mM FAD, and 17 µg target protein solution. Tris-HCl (100 mM, pH 7.4) was added to the reactions, which were then incubated at 25 °C for 20 h. The reactions were terminated by adding 15% KOH/MeOH, and extracted twice with equal volumes of hexane in order to determine the concentration of oxidosqualene formed. The negative control was performed under the same conditions using an extract of *E. coli* cells that carry the empty vector^32^. The HPLC detection method was the same as described above in section 2.2 (“HPLC analysis”).

### Antibody preparation and immunodetection

#### Preparation and titer testing of polyclonal antibodies

New Zealand white rabbits received 400 mg purified protein via subcutaneous injection. Immunizations were performed four times at 2-week intervals. Blood tests were used to determine the titer of the antiserum against the protein using the indirect enzyme-linked immunosorbent assay (ELISA) method. The final preparation of antiserum was conducted when the titer was greater than 1:50,000. Agarose media conjugated to AoSE protein was placed in an antigen affinity purification chromatography column, and a 1:1 mixture of antiserum and PBS was added slowly. The antibody bound to the protein antigen was then eluted with glycine elution buffer to produce the purified antibody. The column eluate was dialysed overnight at 4 °C, followed by determination of purity, concentration, and titer.

#### Determination of polyclonal antibody titer using ELISA

The purified antibody was diluted to 20 µg/mL with 0.05 mol/L carbonate buffer (pH 9.6). Each well of a protein-coated ELISA plate contained 100 µl and was stored overnight at 4 °C. Plates were blocked with 100 µl of 5% skim milk for 1 h at 37 °C. After washing three times with PBS (pH 7.4) containing Tween 20 (PBST), 100 µl of the different dilutions of antiserum were added to each well, and the plate was incubated for 1 h at 37 °C. The negative control consisted of unimmunized serum, and the blank control consisted of PBS solution. After washing three times with PBST, 100 µl goat antibody against horseradish peroxidase (HRP)-conjugated rabbit IgG was added and incubated for 1 h at 37 °C. The substrate TMB was added to each well in a volume of 100 µl and was incubated for 20 min at 37 °C. The reactions were then terminated by adding 50 μL H_2_SO_4_ (2 mol/L) to each well. After the reaction, the absorbance was measured at 450 nm.

#### Determining the specificity of the polyclonal antibody by Western blotting

Total proteins were extracted from leaves, tubers, and roots of *A. orientale* plants using the Plant Total Protein Extraction Kit (BC3720-50T, Solarbio) in different growth stages. Proteins from leaves of *A. orientale* after 0, 1, 2, 3, 4, and 5 days of MeJA treatment were also extracted. The samples were first separated by SDS-PAGE and then transferred to a polyvinylidene fluoride (PVDF) membrane using an electroblotting system. After transfer, the membranes were removed and washed with PBS four times for 5 min each. The membranes were then incubated with blocking solution containing 5% skim milk for 1 h at 37 °C. The primary antibody was diluted with sealing fluid (blocking solution containing 5% skim milk). The membranes were incubated with the primary antibody dilution at 37 °C for 1 h and washed four times for 5 min each. The secondary antibody was also diluted with sealing fluid containing 5% skim milk. Membranes were incubated with the secondary antibody dilution at 37 °C for 1 h; β-Actin was used as a reference. Membranes were washed and developed with enhanced chemiluminescence (ECL) reagent (Santa Cruz Biotechnology), and band densities were quantified using ImageJ software.

### Quantitative RT-PCR analysis of *AoSE* gene expression

Quantitative RT-PCR was performed using the *UBC* gene as an internal standard on an ABI 7500 Real-Time PCR instrument with Toyobo SYBR MIX (QPK-201) to evaluate the relative expression of the *AoSE1* and *AoSE2* genes. Primers were synthesized by GenScript USA, Inc. (UBCF/UBCR, SF7/SR7, and SF8/SR8; Table S1). The cDNAs synthesized from RNA extracted from tubers at different growth stages were used as templates. In addition, to determine the effects of MeJA on the regulation of *AoSE1* and *AoSE2*, the expression of these two genes in MeJA-treated *A. orientale* leaves was also estimated by qRT-PCR. Each 20 µL qRT-PCR reaction consisted of 2 µL cDNA, 1 µL of each primer (10 µM), 10 µL 2X Syber Mix, and 6 µL ddH2O. The amplification conditions for RT-PCR consisted of an initial denaturation step at 95 °C for 10 min, followed by 40 cycles of 95 °C for 10 s and 60 °C for 40 s. The relative expression ratios of the target genes were normalized to the expression of UBC and calculated using the 2^−ΔΔCt^ method.

### Sequence analysis

Sequences were subjected to a BLAST search (NCBI). The largest open reading frames (ORF) were determined using the ORF Finder (www.ncbi.nlm.nih.gov/gorf/). Sequences were then aligned to the *SE* genes of other plant species using Clustal W. The combined full-length cDNAs of the *AoSE* genes were used as queries in BLAST searches. ExPASy online (http://web.expasy.org/compute_pi/) was used to calculate molecular weights and predict isoelectric points. Secondary structures were predicted with PredictProtein (http://www.predictprotein.org/), and tertiary protein structures were predicted with the Phyre2 server (http://www.sbg.bio.ic.ac.uk/phyre2/html). TMHMM (http://www.cbs.dtu.dk/services/TMHMM/) was used to predict transmembrane regions. Signal P4.0 was used to predict signal peptides, and Wolfpsort was used to predict protein subcellular localization. SMART was used to predict the structural domains. A neighbor-joining phylogenetic tree was constructed with MEGA 7 software.

## Results

### Molecular cloning of the full-length *AoSE1* and *AoSE2* cDNAs

Total *A. orientale* leaf RNA was reverse transcribed into cDNA. Using the cDNA as a template, conserved fragments of *SE1* and *SE2* (423 bp) were amplified and cloned using degenerate primers. Based on the nucleotide sequences of *SE1* and *SE2*, 3′- and 5′- RACE specific primers were designed to clone the full-length cDNAs. The 3′- and 5′- RACE fragments of *SE1* were 812 bp and 926 bp in length, respectively. *SE2-*specific DNA fragments of 716 bp and 820 bp were amplified by 3′- and 5′- RACE PCR, respectively. The 5′- and 3′- cDNA fragments were combined with the conserved fragment based on the overlapping regions. From this, we firstly obtained the full-length cDNA clones of the two SE genes, *AoSE1* and *AoSE2*. The full-length *AoSE1* and *AoSE2* cDNAs were 2,157 bp and 1,948 bp, respectively.

**Figure 1:**
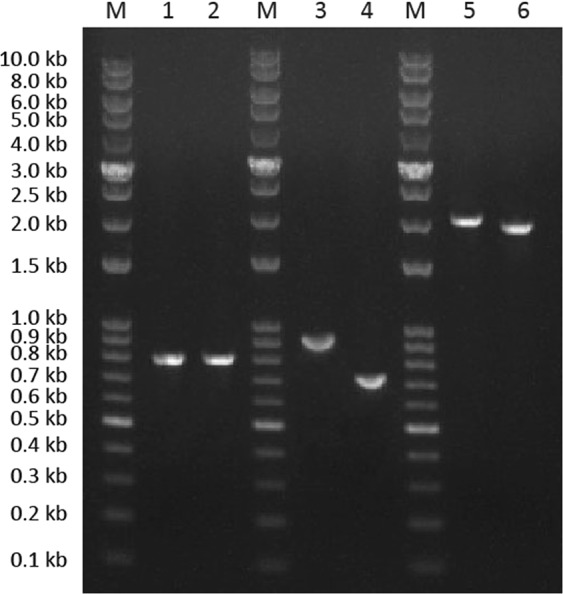
Electropherogram of the *AoSE1* and *AoSE2* cDNA. M: 10000 bp DNA marker, 1: product of *AoSE1* full length cDNA, 2: Product of *AoSE2* full length, 3: product of *AoSE1* 3′-RACE, 4: product of *AoSE2* 3′-RACE, 5: product of *AoSE1* 5′-RACE, 6: product of *AoSE2* 5′-RACE.

To confirm these results, the full-length sequences were amplified using cDNA as a template, followed by sequencing of the products. The results agreed with the DNA sequences of the combined cDNAs (Fig. 1).

### Bioinformatics analysis of *AoSE1* and *AoSE2*

The sequences of the two full-length *AoSE1* and *AoSE2* cDNAs obtained in this study have been deposited in the Genbank database under accession numbers KU856661 and KU856662. The cDNA sequence of *AoSE1* encodes a predicted protein of 520 amino acid residues with a molecular weight of 56.38 kDa. The full-length *AoSE2* cDNA encodes a protein of 521 amino acids and a calculated molecular mass of 56.15 kDa.

**Figure 2:**
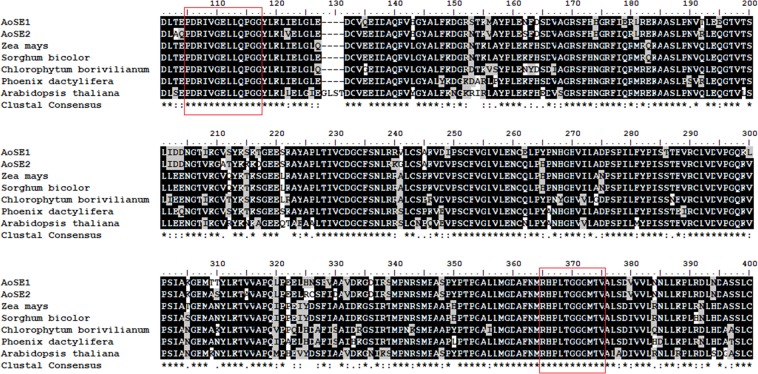
Amino acid sequences of AoSEs are aligned with those from the other plants using Clustal W multiple sequence alignment tool. The symbol indicates that the aligned residues are identical. Conserved or semi-conserved substitutions are marked by (:) and (.), respectively. The two conserved domains are numbered and indicated in the box.

The two sequences shared 86.92% amino acid homology and were compared with proteins from other species by BLAST (Fig. 2). Multiple amino acid sequence alignments revealed that AoSE1 and AoSE2 were 82.23%, 77.88%, 77.5%, 75.96%, 70.96% and 82.85%, 78.69%, 77.16%, 76.73%, and 71.40% homolgous with similar proteins from *Zea mays* (maize), *Sorghum bicolor* (sorghum), *Phoenix dactylifera* (date palm), *Chlorophytum borivilianum* (safed musli; Asparagaceae), and *Arabidopsis thaliana* (thale cress), respectively.

We used TMHMM to predict possible transmembrane regions in the sequences of AoSE1 and AoSE2. The AoSE1 protein is predicted to contain one transmembrane region, which is located at amino acids 2–24. The AoSE2 protein contains four predicted transmembrane regions located at amino acids 3–23, 57–77, 451–471, and 479–499. In addition, functional domain analysis revealed that AoSE1 and AoSE2 both contain a FAD-binding 3 domain, a GIDA domain, a NAD binding 8 domain, and an SE domain. The results of analysis by the online Signal P4.0 Server show that AoSE1 has no signal peptide, while AoSE2 is predicted to have one signal peptide. WOLFPSORT prediction shows that AoSE1 is most likely to be an integral membrane protein, while AoSE2 is most likely to be located in the endoplasmic reticulum because the AoSE2 sequence contains a signal peptide.

The structure of plant SE proteins has not yet been analyzed. However, the crystal structure of the catalytic domain of the human squalene epoxidase protein (6C6N.1.A), which also belongs to the SE superfamily, has been identified by X-ray diffraction, so the human SQLE protein was used as a template to predict the 3D structures of AoSE1 and AoSE2. The AoSE1 and AoSE2 catalytic domains have a critical region that controls oxygen transfer, and the enzymatic reactions happen in this structure.

MEGA 7 software was used to construct a neighbor-joining phylogenetic tree. SE protein sequences from other plant species available in GenBank were compared with those of *A. orientale* through cluster analysis (Fig. S2). Proteins from *A. orientale* clustered with those from *Z. mays, C. borivilianum*, *Avena strigosa*, and *Sorghum bicolor* on the monocotyledon branch. Fifteen other SE proteins clustered on the dicotyledon branch.

### Prokaryotic expression of AoSE1 and AoSE2

**Figure 3:**
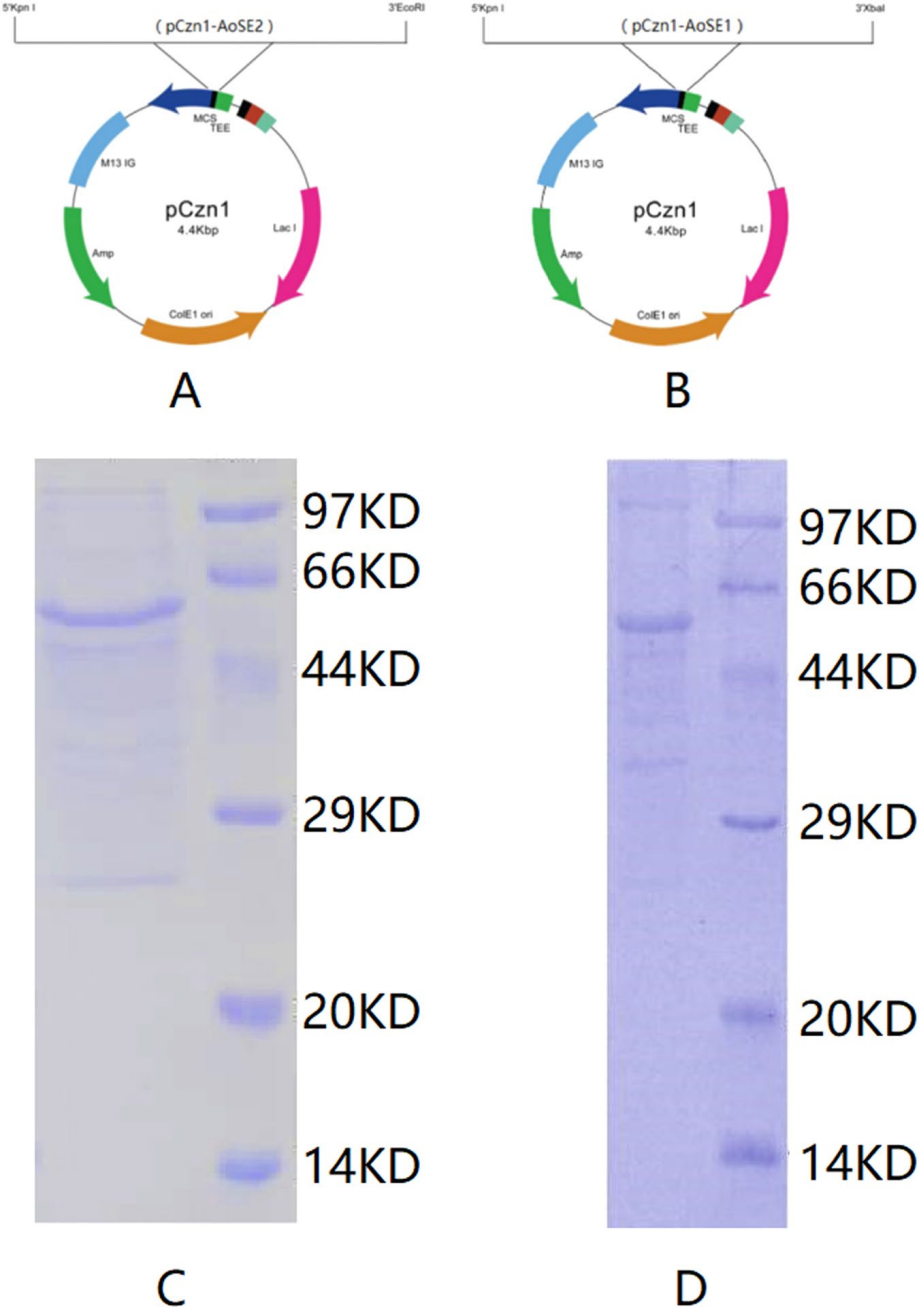
Prokaryotic expression of AoSE1 and AoSE2. (**A**) Construction of the prokaryotic expression vector for AoSE2, (**B**) construction of the prokaryotic expression vector for AoSE1, (**C**) AoSE1 protein purification analysis. Lane M: protein marker; lane 1: purified AoSE1. (**D**) AoSE2 protein purification analysis. Lane M: protein marker; lane 1: purified AoSE2.

We performed prokaryotic expression in *E. coli* to obtain purified AoSE1 and AoSE2 proteins, which were used to explore their biological functions. Using the PCR guide for restriction enzyme cutting sites, we inserted the *AoSE1* cDNA into the pCzn1 expression vector between the KpnI and XbaI restriction sites to give a recombinant expression vector containing the His6-tag, pCzn1-AoSE1 (Fig. 3A). The plasmid was extracted and digested with KpnI and XbaI to give a 1500 bp DNA fragment as predicted. We also cloned the *AoSE2* cDNA into the pCzn1 expression vector between the KpnI and EcoRI restriction sites to give pCzn1-AoSE2, a recombinant expression vector containing the His6-tag (Fig. 3B). This plasmid also gave a 1500 bp DNA fragment after digestion with KpnI and EcoRI.

DNA sequencing showed that the *AoSE* sequences inserted into the pCzn1-AoSE1 and pCzn1-AoSE2 vectors were identical to those of the cloned genes, *AoSE1* and *AoSE2*. This indicated that the recombinant prokaryotic expression vectors, pCzn1-AoSE1 and pCzn1-AoSE2, were constructed correctly and should produce His6-tag fusion proteins in *E. coli*.

The results of SDS-PAGE showed that the molecular weights of the His6-tag fusion proteins were approximately 58.73 kDa and 58.11 kDa for AoSE1 and AoSE2, respectively, which is consistent with the predicted sizes. When the *E. coli* cultures harboring the recombinant plasmids were induced with 0.2 mM IPTG for 12 h at 11 °C, the fusion proteins could be detected in the cell pellets, indicating that the recombinant proteins were present as inclusion bodies. Accordingly, we had to denature, renature, and then resolubilize the target proteins in order to purify them. The purified proteins were obtained through dilution and dialysis (Fig. 3C,D).

### Functional identification of AoSE1 and AoSE2

**Figure 4:**
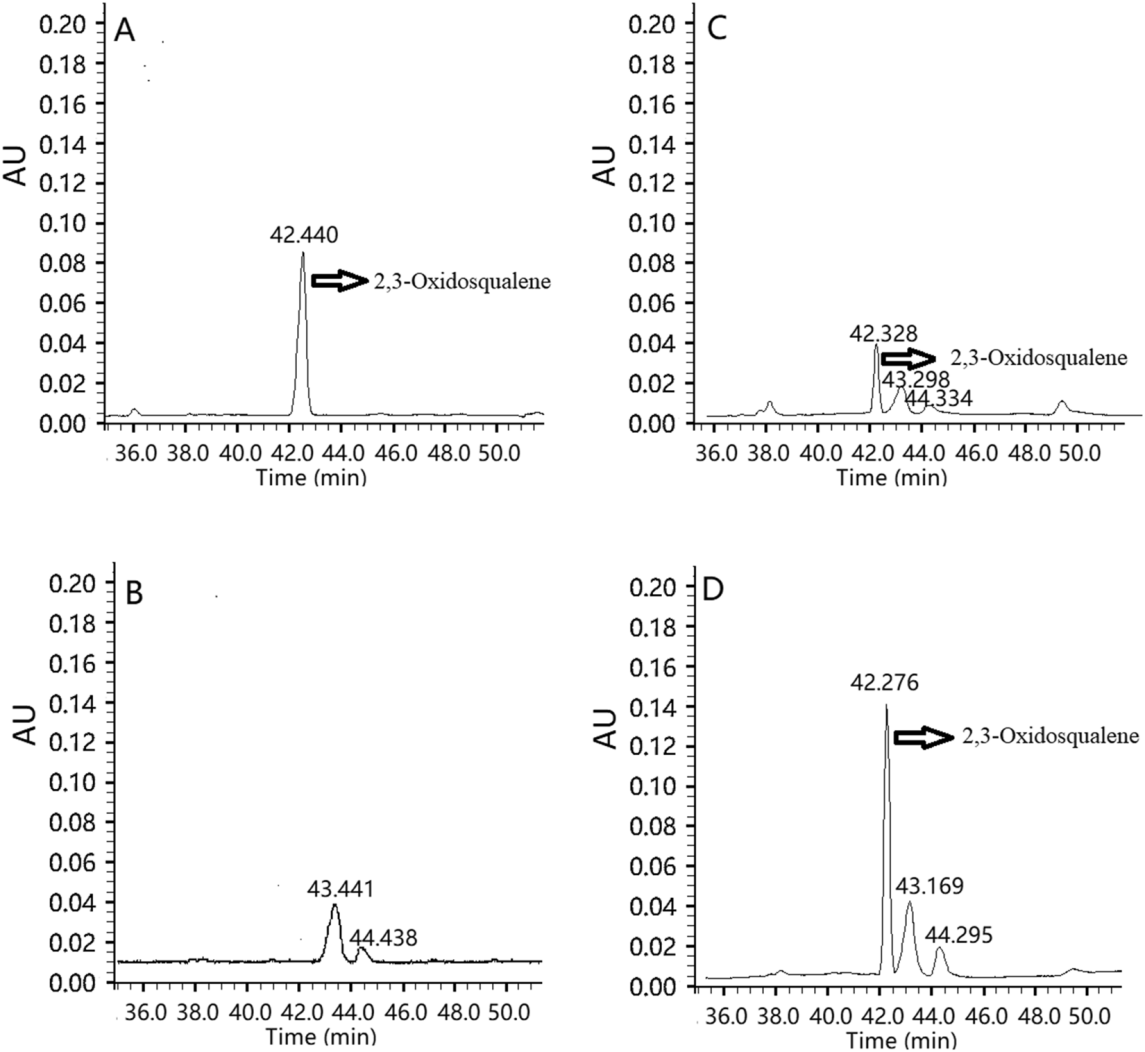
HPLC chromatograms of AoSE1 and AoSE2 enzymatic reactions *in vitro*. (**A**) Standard 2,3- oxidosqualene; (**B**) the control group; (**C**) the reaction products of AoSE1; (**D**) the reaction products of AoSE2.

To biochemically characterize the enzymatic activities of AoSE1 and AoSE2, we established an *in vitro* enzymatic assay system. In the HPLC analysis, the peak at 42.4 min in the chromatogram corresponds to that observed for authentic 2,3-oxidosqualene, while no such peak was detected in the vector-control (Fig. 4). These results showed that both AoSE1 and AoSE2 can catalyze the conversion of squalene to 2,3-oxidosqualene. Based on the amount of the 2,3-oxidosqualene product in the reactions, the catalytic function of AoSE2 is obviously stronger than that of AoSE1.

### Antibody preparation and immunodetection of AoSE1 and AoSE2

**Figure 5:**
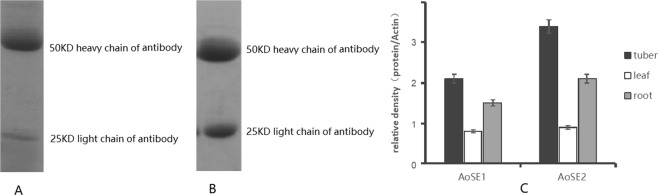
Antibody preparation and immunodetection of AoSE1 and AoSE2. (**A**) SDS-PAGE of purified AoSE1 antibody; (**B**) SDS-PAGE of purified AoSE2 antibody; (**C**) the expression levels of protein AoSE1 and AoSE2 in tubers, leaves, and roots.

We used an immunodetection method to determine whether the expression of AoSE1 and AoSE2 is related to the alisol B 23-acetate content. Antibodies were produced in New Zealand white rabbits immunized with the purified fusion proteins. The specificities of the anti-AoSE1/2 IgG antibodies were determined by ELISA, and the concentrations were estimated using a BCA protein concentration kit. The antibody concentrations measured by the BCA method were 0.68 mg/mL (anti-AoSE1) and 0.9 mg/mL (anti-AoSE2). These results indicated that the anti-AoSE antibodies had a high degree of detectable sensitivity at a concentration of 0.1 mg/ml. After electro-elution, the antibody purity was >95% (Fig. 5A,B). ELISA results revealed that the titer of the polyclonal antibodies directed against AoSE1 and AoSE2 was very high, with dilutions greater than 1:512,000.

Western blots were performed using rabbit antiserum as the primary antibody to determine whether AoSE1 and AoSE2 could be identified in plants with polyclonal antibodies. The results showed that the antibodies successfully detected the presence of AoSE1 and AoSE2 in different organs of *A. orientale*. Studies were only conducted on vegetative organs known to be relevant for traditional medicine, so we measured the AoSE1 and AoSE2 levels in tubers, leaves, and roots. AoSE1 and AoSE2 showed the strongest expression in tubers, with lower levels in leaves and roots, and the levels of AoSE2 were slightly higher than the levels of AoSE1 (Figs. S3, 5C).

### Gene expression and protein levels of AoSE1 and AoSE2 during different growth stages in *A*. *orientale*

**Figure 6:**
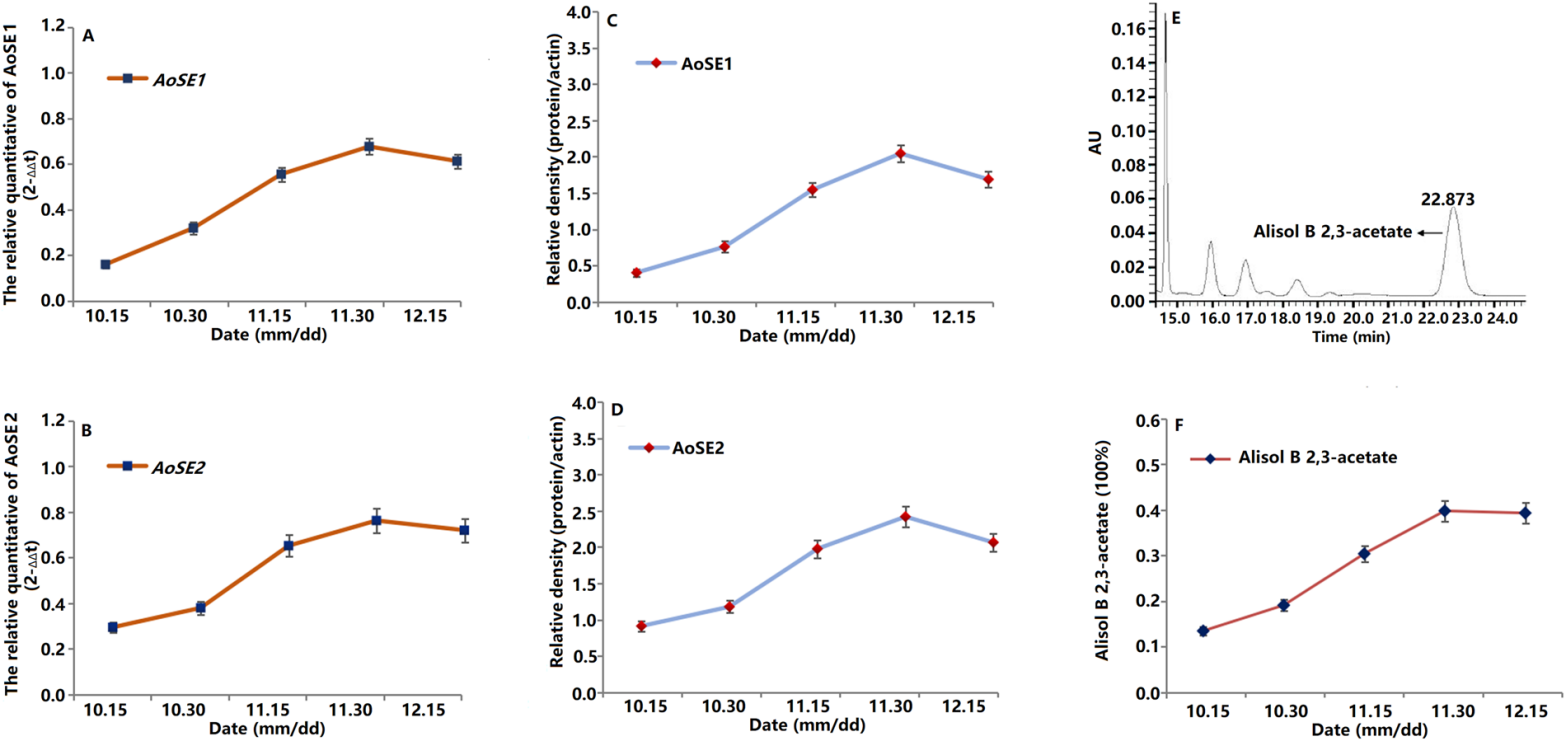
Samples were taken from *A. orientale* tubers from October 15 to December 15. (**A**,**B**) Relative quantitation of the *AoSE1 and AoSE2* transcript at different growth phases in *A.orientale*; (**C**,**D**) western blot analysis demonstrated the AoSE1 and AoSE2 signal at different growth phases in *A.orientale*; (**E**) HPLC chromatograms of alisol B 23-acetate in *A. orientale*; (**F**) content of alisol B 23-acetate at different growth phases in *A.orientale*.

In order to understand the pattern of AoSE1 and AoSE2 expression during the growth of *A. orientale*, the relative mRNA levels of *AoSE1* and *AoSE2*, and the corresponding protein levels in the tubers were assayed by qRT-PCR and Western blotting, respectively (Fig. 6A–D). The relative expression of the *AoSE1* and *AoSE2* genes increased from October to early December, and reached their maximum at the end of November. Similarly, the relative AoSE1 and AoSE2 protein levels increased from October and started to decline in early December.

### Analysis of active components during different growth stages of *A*. *orientale*

Alisol B 23-acetate is the main active component in *A. orientale* and is used as a general index to evaluate quality. The content of alisol B 23-acetate in tubers was determined by HPLC. We were able to calculate the concentration of alisol B 23-acetate in tubers at different growth stages using the concentration of the standard compound as the control. Results showed that the alisol B 23-acetate level gradually increased during growth, reaching its maximum of 2.4-fold at the end of November compared to the level in early October, which mirrored the expression profile of the *AoSE1* and *AoSE2* genes (Fig. 6F).

The alisol B 23-acetate content increased gradually from October to November, and was constant until December. Only a slight decrease was observed during the early part of December. The correlation between AoSE1 and AoSE2 levels and alisol B 23-acetate content was analyzed by SPSS19.0. Statistical analysis revealed a positive correlation at a significance level of 0.05 (Table S2).

### Effects of MeJA treatment on AoSE1 and AoSE2 expression and the levels of active components in *A*. *orientale*

**Figure 7:**
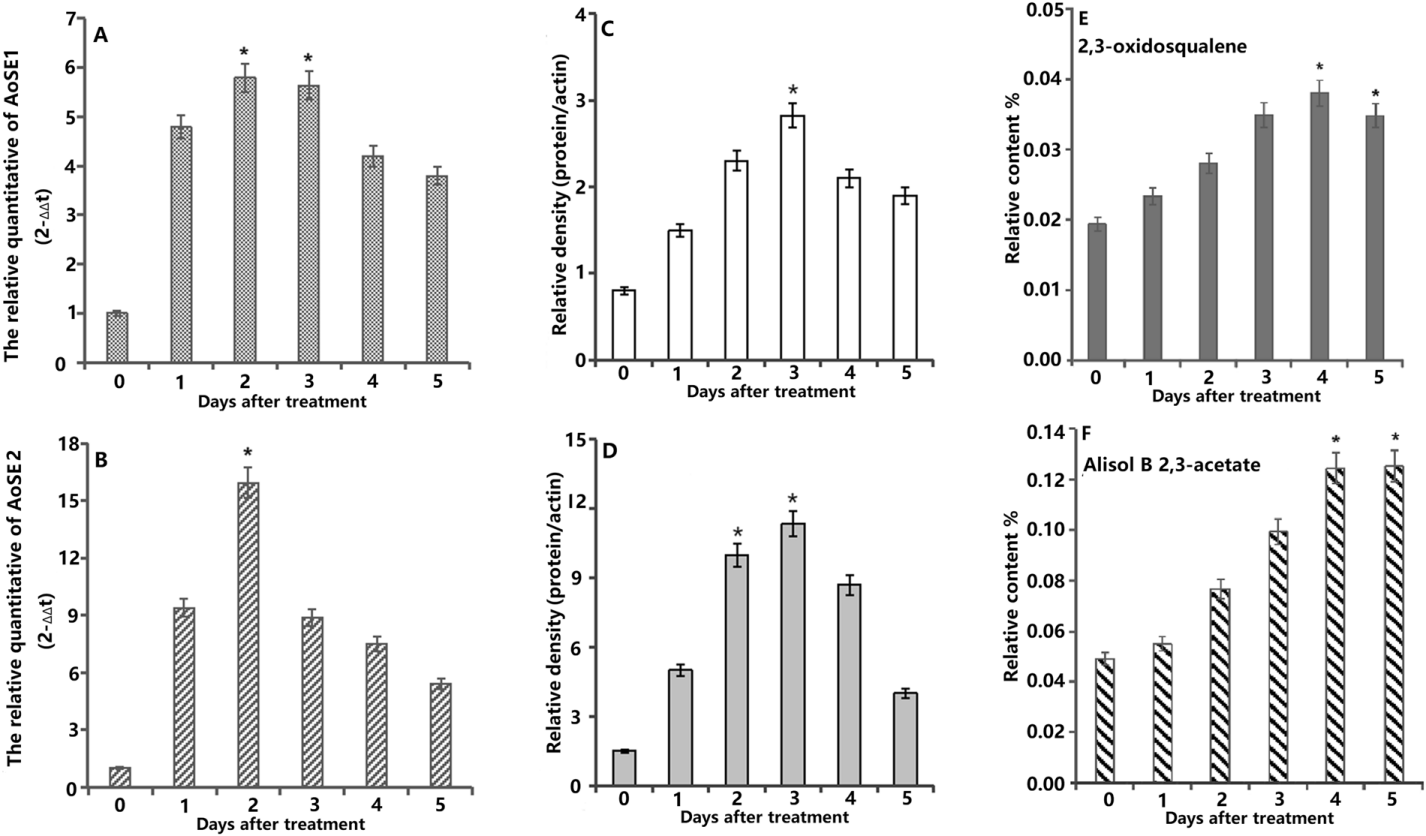
Effects of MeJA on AoSE1, AoSE2 expressions and 2,3-oxidosqualene, alisol B 23-acetate contents. (**A**,**B**) effects of MeJA on *AoSE1 and AoSE2* genes expressions; (**C**,**D**) effects of MeJA on AoSE1 and AoSE2 proteins expressions; (**E**) effects of MeJA on accumulation of 2,3-oxidosqualene contents; (**F**) effects of MeJA on accumulation of alisol B 23-acetate contents in *A.orientale*. *P < 0.05.

Based on the previous results, we conducted a MeJA induction experiment. The effects of the elicitor (MeJA) on AoSE1 and *AoSE2* expression, the contents of 2,3-oxidosqualene and alisol B 23-acetate, and the fresh weights of *A. orientale* plants are shown in Fig. 7 and Table S3.

The relative transcriptional levels of *AoSE1* and *AoSE2* reached a maximum on the third day after MeJA treatment, with increases of 5.84-fold (*AoSE1*) and 15.93-fold (*AoSE2*) compared to the control group (Fig. 7A,B). In response to MeJA treatment, the expression of both AoSE1 and AoSE2 at the translational level increased gradually and reached the maximum on the third day, 3.53-fold and 7.57-fold higher than the control group, respectively (Fig. 7C,D).

The 2,3-oxidosqualene and alisol B 23-acetate levels were also significantly increased by MeJA treatment. The 2,3-oxidosqualene content increased 1.96-fold on the fourth day after treatment, and began to decrease on the fifth day after treatment (Fig. 7E). These results showed that there were similar trends for the alisol B 23-acetate and 2,3-oxidosqualene contents in response to MeJA treatment. The Alisol B 23-acetate level peaked on the fourth day after treatment, with an increase of 2.53-fold, and it began to decrease on the fifth day after treatment (Fig. 7F).

During the 5-day MeJA induction experiment, both the treatment and control groups showed no significance differences in fresh weight (P > 0.05) (Table S3).

The correlations between the mRNA, protein, and alisol B 23-acetate levels in *A. orientale* treated with MeJA at different time points was analyzed using SPSS19.0. Statistical analysis revealed a positive correlation at a significance level of 0.05 (Table S4).

## Discussion

Protostane triterpenes are natural plant products with distinct pharmacological activities, but their natural distribution is limited to a few plant families^33,34^. Squalene epoxidase (SE) is the rate-limiting enzyme in the triterpene biosynthesis pathway.

In this study, two *SE* genes, *AoSE1* and *AoSE2* were firstly cloned from leaves of *A. orientale*. There is no predicted signal peptide in AoSE1, indicating that AoSE1 is inserted into the endoplasmic reticulum in a Golgi-independent manner^16,35^. The AoSE2 protein is predicted to have one signal peptide, indicating that it is transported after being synthesized in the cytoplasm.

The predicted protein sequences of AoSE1 and AoSE2 from *A. orientale* differ from other plant SE protein sequences, but the functional domains are similar to those found in other SEs. Functional domain analysis revealed that AoSE1 and AoSE2 both contain a FAD-binding 3 domain, a GIDA domain, a NAD-binding 8 domain, and an SE domain. A gene for *SE* was first cloned from *Saccharomyces cerevisiae* (baker’s yeast) where it was named *ERG1*^36^. This protein contains a putative FAD binding site and has squalene monooxygenase activity^37,38^. Putative transmembrane regions are present in the C terminal region of the protein. An SE domain and two FAD/NAD-binding domains are critical to the normal function of SE enzymes^39^, and the results of the domain analysis indicate that the SE proteins characterized in our study have catalytic activity to convert squalene to 2,3-oxidosqualene. The AoSE1 protein contains one transmembrane region and AoSE2 contains four transmembrane regions; both proteins have SE catalytic activity and are similar to other SEs from *P. ginseng*, *A. senticosus*, and other plant species^22,36^.

In neighbor-joining phylogenetic tree, AoSE1 and AoSE2 were found to be more closely related to the SEs from *C. borivilianum*, *A. strigosa*, and *Z. mays* than other species and they formed a distinct monocotyledon clade^22,40,41^. This shows that the degree of sequence homology is closely related to the evolutionary relationships. The phylogenetic analysis of amino acid sequences is helpful to understand the evolutionary trajectory of plants and is of great significance for further understanding of gene function.

Because the low level of expression of SE in plants limits its functional identification, we expressed the AoSE1 and AoSE2 proteins in *E. coli* so that we could explore their functions in protostane triterpenes biosynthesis. SE is an endoplasmic reticulum (ER) membrane-bound protein, and it usually accumulates in the form of inclusion bodies when expressed in *E. coli* cells^42^. To enhance its solubility, the transmembrane domains of the natural AoSE1 and AoSE2 protein sequences must be truncated prior to expression in *E. coli*. We used 8 mol/L urea to reduce precipitation in the renaturation process, ensuring that the inclusion body proteins formed the correct disulfide bonds, resulting in AoSE1 and AoSE2 proteins with biological activity. AoSE1 and AoSE2 were successfully purified as His-tag fusion proteins and were used in functional enzymatic activities *in vitro*, the results of which showed that both AoSE1 and AoSE2 are able to convert squalene to 2,3-oxidosqualene. This suggests that AoSE proteins are responsible for the biosynthesis of alisol B 23-acetate *in vivo*, because triterpene skeleton is synthesised from the important precursor 2,3-oxidosqualene^43^. These results provide a basis for further research on using the regulation of *AoSE1* and *AoSE2* expression to increase the content of protostane triterpenes in *A. orientale*.

We used rapid immunodetection of AoSE1 and AoSE2 to solve the difficulty of analyzing the expression of AoSE proteins in plants. Western blot analysis showed that the polyclonal antibodies specifically recognized AoSE1 and AoSE2 in *A. orientale* with good sensitivity and also confirmed the reliability of the immunoassay.

We found that AoSE protein levels were the highest in the tubers among the tested tissues of mature *A. orientale* during the collection period, suggesting that tubers could be the major site of synthesis of protostane triterpenes in *A. orientale*. This was consistent with the fact that *A. orientale* tubers contain the highest content of protostane triterpenes and have great medicinal value^44^. Tissue-specific expression could be used as a mechanism to regulate the supply of MVA (mevalonic acid) to the biosynthetic pathways localized in these plant organs.

We also wanted to determine whether the levels of AoSE1 and AoSE2 were correlated with the contents of the active constituents at different stages of growth. For plants collected in the wild, both the mRNA and protein levels of the AoSEs increased from October to November and decreased slightly in December. The contents of the active components showed similar trends compared with *AoSE* gene expression and protein levels. The synthesis of secondary metabolites peaked at the end of November, after which synthesis decreased in *A. orientale* due to the onset of senescence and a reduction in metabolic rate. The significant positive correlation between the levels of AoSE1 and AoSE2 and protostane triterpenes further confirms that these two enzymes are the key points in the protostane triterpenes biosynthesis pathway in *A. orientale*.

MeJA is the main elicitor of triterpene biosynthesis in plants^45^. Based on our experimental data, we found that MeJA treatment can induce *AoSE1* and *AoSE2* gene expression and increase AoSE1 and AoSE2 protein levels, and it also elicits the synthesis of 2,3-oxidosqualene and alisol B 23-acetate. The contents of 2,3-oxidosqualene and alisol B 23-acetate were increased by 1.96- and 2.53-fold on the fourth day, respectively, compared with the untreated control. Elicitor induction is considered an effective way to improve the yields of secondary metabolites that function in the communication of plants with their environment by participating in either extracellular or intracellular signal transduction^46,47^. A previous study showed that, MeJA induced triterpene biosynthesis by regulating the expression of genes including the transcription factors (TFs)^45,48,49^. TFs are sequence-specific DNA-binding proteins that interact with the promoter regions of target genes to modulate their expression^50^. The WRKY, MYC and MYB TFs were shown as regulators of triterpene biosynthesis in plants in response to MeJA^51,52^. The PqWRKY1 is a positive regulator related to triterpene ginsenoside biosynthesis in *Panax quinquefolius*^53^. The expression levels of WsMYC2 was enhanced and the contents of withanolides in the plants also increased significantly in *Withania somnifera* (L.) Dunal after MeJA treatment^54^. In * P. ginseng*, PgMYB2 could up-regulate the expression of dammarenediol synthase after MeJA treatment^55^. Moreover, the increase in membrane permeability by MeJA application can facilitate the combination of substrate and enzyme, and increase the rate of secondary metabolite synthesis^56^. In a study of the effects of MeJA in *Betula platyphylla*, it was found that the expression of all *SE* genes were increased^57^. Thus, it is possible that the increased protostane triterpene content observed in our study may be due to the activation of genes for key isoprenoid pathway enzymes by MeJA. The positive correlation between the mRNA, protein, and alisol B 23-acetate contents at different times following MeJA treatment implies that MeJA increased the alisol B 23-acetate content by inducing the expression of *AoSE1* and *AoSE2*. Although exogenous elicitors do not directly participate in the synthesis of secondary metabolites, they can activate the expression of related genes in plants, leading to the accumulation of secondary metabolites^58^.

The results presented here suggest that AoSE1 and AoSE2 function as the key points in the regulation of protostane triterpenes biosynthesis in *A. orientale*, and that MeJA can regulate the biosynthetic efficiency of these compounds by inducing the expression of *AoSE1* and *AoSE2*.

This work supports the involvement of SE genes in the triterpene biosynthesis pathway in *A. orientale*, and contributes to an improved understanding of the regulation of the biosynthesis of these bioactive compounds by MeJA *in vivo*.

## Supplementary information

supplement material

## References

[1] Wang H, Shi S, Wang S (2018). Can highly cited herbs in ancient Traditional Chinese medicine formulas and modern publications predict therapeutic targets for diabetes mellitus?. J Ethnopharmacol.

[2] Li Sen, Wang Lu, Du Zhifeng, Jin Shuna, Song Chengwu, Jia Shuailong, Zhang Yang, Jiang Hongliang (2019). Identification of the lipid-lowering component of triterpenes from Alismatis rhizoma based on the MRM-based characteristic chemical profiles and support vector machine model. Analytical and Bioanalytical Chemistry.

[3] Huang MQ, Xu W, Wu SS, Lu JJ, Chen XP (2013). A 90-day subchronic oral toxicity study of triterpene-enriched extract from Alismatis Rhizoma in rats. Food Chem Toxicol.

[4] Jang MK (2015). Protective Effects of Alisma orientale Extract against Hepatic Steatosis via Inhibition of Endoplasmic Reticulum Stress. Int J Mol Sci.

[5] Mai ZP (2015). Protostane Triterpenoids from the Rhizome of Alisma orientale Exhibit Inhibitory Effects on Human Carboxylesterase 2. J Nat Prod.

[6] Meng Q (2015). Protective effects of alisol B 23-acetate from edible botanical Rhizoma alismatis against carbon tetrachloride-induced hepatotoxicity in mice. Food Funct.

[7] Kurapati, K. R. V., Atluri, V. S., Samikkannu, T., Garcia, G. & Nair, M. P. N. Natural Products as Anti-HIV Agents and Role in HIV-Associated Neurocognitive Disorders (HAND): A Brief Overview. *Front Microbiol* **6**, 10.3389/fmicb.2015.01444 (2016). 10.3389/fmicb.2015.01444 PMC4709506 26793166

[8] Li S (2016). The metabolic change of serum lysophosphatidylcholines involved in the lipid lowering effect of triterpenes from Alismatis rhizoma on high-fat diet induced hyperlipidemia mice. J Ethnopharmacol.

[9] Zhang LL (2017). Therapeutic potential of Rhizoma Alismatis: a review on ethnomedicinal application, phytochemistry, pharmacology, and toxicology. Ann N Y Acad Sci.

[10] Tian T, Chen H, Zhao YY (2014). Traditional uses, phytochemistry, pharmacology, toxicology and quality control of Alisma orientale (Sam.) Juzep: a review. J Ethnopharmacol.

[11] Wang C (2004). Reversal of P-glycoprotein-mediated multidrug resistance by Alisol B 23-acetate. Biochem Pharmacol.

[12] Galanie S, Thodey K, Trenchard IJ, Interrante MF, Smolke CD (2015). Complete biosynthesis of opioids in yeast. Science.

[13] Wu G (2016). Metabolic Burden: Cornerstones in Synthetic Biology and Metabolic Engineering Applications. Trends Biotechnol.

[14] Chen CC, Liang CS, Kao AL, Yang CC (2010). HHP1, a novel signalling component in the cross-talk between the cold and osmotic signalling pathways in Arabidopsis. J. Exp. Bot..

[15] Xiang Y (2011). A jacalin-related lectin-like gene in wheat is a component of the plant defence system. J. Exp. Bot..

[16] Vranova E, Coman D, Gruissem W (2013). Network analysis of the MVA and MEP pathways for isoprenoid synthesis. Annu Rev Plant Biol.

[17] Gill S, Stevenson J, Kristiana I, Brown AJ (2011). Cholesterol-Dependent Degradation of Squalene Monooxygenase, a Control Point in Cholesterol Synthesis beyond HMG-CoA Reductase. Cell Metab.

[18] Huijbers MME, Montersino S, Westphal AH, Tischler D, Van Berkel WJH (2014). Flavin dependent monooxygenases. Arch Biochem Biophys.

[19] He FM, Zhu YP, He MX, Zhang YZ (2008). Molecular cloning and characterization of the gene encoding squalene epoxidase in Panax notoginseng. DNA Seq..

[20] Xu, J. *et al*. Panax ginseng genome examination for ginsenoside biosynthesis. *Gigascience* **6**, 10.1093/gigascience/gix093 (2017). 10.1093/gigascience/gix093 PMC5710592 29048480

[21] Han JY, In JG, Kwon YS, Choi YE (2010). Regulation of ginsenoside and phytosterol biosynthesis by RNA interferences of squalene epoxidase gene in Panax ginseng. Phytochemistry.

[22] Zhu L, Ma LQ, Xu F, Yan XJ, Zhang WW (2017). Cloning and Expression Analysis of a Squalene Epoxidase Gene from Ginkgo biloba. Notulae Botanicae Horti Agrobotanici Cluj-Napoca.

[23] Yuan SX (2013). Gene cloning of squalene synthase in Alisma orientalis and its bioinformatic analysis. Chinese Traditional and Herbal. Drugs.

[24] Gu W (2015). Characterization and function of the 3-hydroxy-3-methylglutaryl-CoA reductase gene in Alisma orientale (Sam.) Juz. and its relationship with protostane triterpene production. Plant Physiol Biochem.

[25] Liu QZ (2017). Prokaryotic expression, functional identification of squalene synthase in Alisma orientale and its immunoassay study. Zhongguo Zhong Yao Za Zhi.

[26] Westfall CS, Muehler AM, Jez JM (2013). Enzyme Action in the Regulation of Plant Hormone Responses. J Biol Chem.

[27] Ji Y, Liu J, Xing D (2016). Low concentrations of salicylic acid delay methyl jasmonate-induced leaf senescence by up-regulating nitric oxide synthase activity. J. Exp. Bot..

[28] Zhou M, Memelink J (2016). Jasmonate-responsive transcription factors regulating plant secondary metabolism. Biotechnol Adv.

[29] Kim YS, Yeung EC, Hahn EJ, Paek KY (2007). Combined effects of phytohormone, indole-3-butyric acid, and methyl jasmonate on root growth and ginsenoside production in adventitious root cultures of Panax ginseng C.A. Meyer. Biotechnol Lett.

[30] Gonçalves Sandra, Romano Anabela (2018). Production of Plant Secondary Metabolites by Using Biotechnological Tools. Secondary Metabolites - Sources and Applications.

[31] Jieshu W (2013). Regulatory Effect of Methyl Jasmonate on HMGR, DXR and DXS Genes Expression in Amomum villosum Lour. Journal of Guangzhou University of Traditional Chinese Medicine.

[32] Shanmugabalaji V (2018). Chloroplast biogenesis controlled by DELLA-TOC159 interaction in early plant development. Current Biology.

[33] Zhao M, Godecke T, Gunn J, Duan JA, Che CT (2013). Protostane and Fusidane Triterpenes: A Mini-Review. Molecules.

[34] Xin XL (2018). Two new protostane-type triterpenoids from Alisma orientalis. Natural Product Research.

[35] Nickel W, Seedorf M (2008). Unconventional mechanisms of protein transport to the cell surface of eukaryotic cells. Annu Rev Cell Dev Biol.

[36] Zhou J (2018). Functional characterization of squalene epoxidase genes in the medicinal plant Tripterygium wilfordii. International Journal of Biological Macromolecules.

[37] Ruckenstuhl C, Eidenberger A, Lang S, Turnowsky F (2005). Single amino acid exchanges in FAD-binding domains of squalene epoxidase of Saccharomyces cerevisiae lead to either loss of functionality or terbinafine sensitivity. Biochem Soc Trans.

[38] Heine T (2017). Engineering Styrene Monooxygenase for Biocatalysis: Reductase-Epoxidase Fusion Proteins. Appl Biochem Biotechnol.

[39] Padyana, A. K. *et al*. Structure and inhibition mechanism of the catalytic domain of human squalene epoxidase. *Nature Communications* **10**, 10.1038/s41467-018-07928-x (2019). 10.1038/s41467-018-07928-x PMC6327030 30626872

[40] Kalra S, Kumar S, Singh K (2014). Molecular analysis of squalene epoxidase gene from Chlorophytum borivilianum (Sant. and Fernand.). J Plant Biochem Biotechnol.

[41] Dong L (2018). Co-expression of squalene epoxidases with triterpene cyclases boosts production of triterpenoids in plants and yeast. Metabolic Engineering.

[42] Pollier J (2019). A widespread alternative squalene epoxidase participates in eukaryote steroid biosynthesis. Nature Microbiology.

[43] Bach TJ (2017). Secondary Metabolism: High cholesterol in tomato. Nature. Plants.

[44] GU W (2011). Molecular cloning of farnesyl pyrophosphate synthase from Alisma orientale (Sam.) Juzep. and its distribution pattern and bioinformatics analysis. Acta Pharmaceutica Sinica.

[45] Li MJ, Liu MY, Peng FT, Fang L (2015). Influence factors and gene expression patterns during MeJa-induced gummosis in peach. J Plant Physiol.

[46] Murata Y, Mori IC, Munemasa S (2015). Diverse stomatal signaling and the signal integration mechanism. Annu Rev Plant Biol.

[47] Sandor R (2016). Plasma membrane order and fluidity are diversely triggered by elicitors of plant defence. J. Exp. Bot..

[48] Wasternack C, Hause B (2013). Jasmonates: biosynthesis, perception, signal transduction and action in plant stress response, growth and development. Annals of Botany.

[49] Jiang YF, Ye JY, Li S, Niinemets U (2017). Methyl jasmonate-induced emission of biogenic volatiles is biphasic in cucumber: a high-resolution analysis of dose dependence. J. Exp. Bot..

[50] Franco-Zorrilla JM (2014). DNA-binding specificities of plant transcription factors and their potential to define target genes. Proc. Natl Acad. Sci. USA.

[51] Mishra S (2013). Wound induced tanscriptional regulation of benzylisoquinoline pathway and characterization of wound inducible PsWRKY transcription factor from Papaver somniferum. PLoS ONE.

[52] Misra RC (2014). Methyl jasmonate-elicited transcriptional responses and pentacyclic triterpene biosynthesis in sweet basil. Plant Physiol.

[53] Sun Y (2013). Discovery of WRKY transcription factors through transcriptome analysis and characterization of a novel methyl jasmonate-inducible PqWRKY1 gene from Panax quinquefolius. Plant Cell Tissue Organ Cult..

[54] Sharma A, Rather GA, Misra P, Dhar MK, Lattoo SK (2019). Jasmonate responsive transcription factor WsMYC2 regulates the biosynthesis of triterpenoid withanolides and phytosterol via key pathway genes in Withania somnifera (L.) Dunal. Plant Mol Biol.

[55] Liu T (2019). PgMYB2, a MeJA-Responsive Transcription Factor, Positively Regulates the Dammarenediol Synthase Gene Expression in Panax Ginseng. Int J Mol Sci.

[56] Lu M (2016). AtCNGC2 is involved in jasmonic acid-induced calcium mobilization. J. Exp. Bot..

[57] Zhang M (2016). Molecular cloning and promoter analysis of squalene synthase and squalene epoxidase genes from Betula platyphylla. Protoplasma.

[58] Lyons R, Manners JM, Kazan K (2013). Jasmonate biosynthesis and signaling in monocots: a comparative overview. Plant Cell Reports.

