# Supplementary material for: Methyl jasmonate promote protostane triterpenes accumulation by up-regulating the expression of squalene epoxidases in Alisma orientale
Source: Sci Rep. 2019 Dec 2;9:18139. doi: 10.1038/s41598-019-54629-6 (PMC6889204; doi:10.1038/s41598-019-54629-6)
Supplement: Supplementary file 1 — supplement material [file 41598_2019_54629_MOESM1_ESM.pdf]

Methyl jasmonate promote protostane triterpenes accumulation by up-regulating the expression of squalene epoxidases in *Alisma orientale*

Tian Rong[1], Gu Wei[1*], Gu Yuchen[1], Geng Chao[1], Xu Fei[1], Wu Qinan[1], Chao Jianguo[1], Xue Wenda[1], Zhou Chen[1], Wang Fan[1]

[1] School of Pharmacy, Nanjing University of Chinese Medicine, Nanjing 210023, China

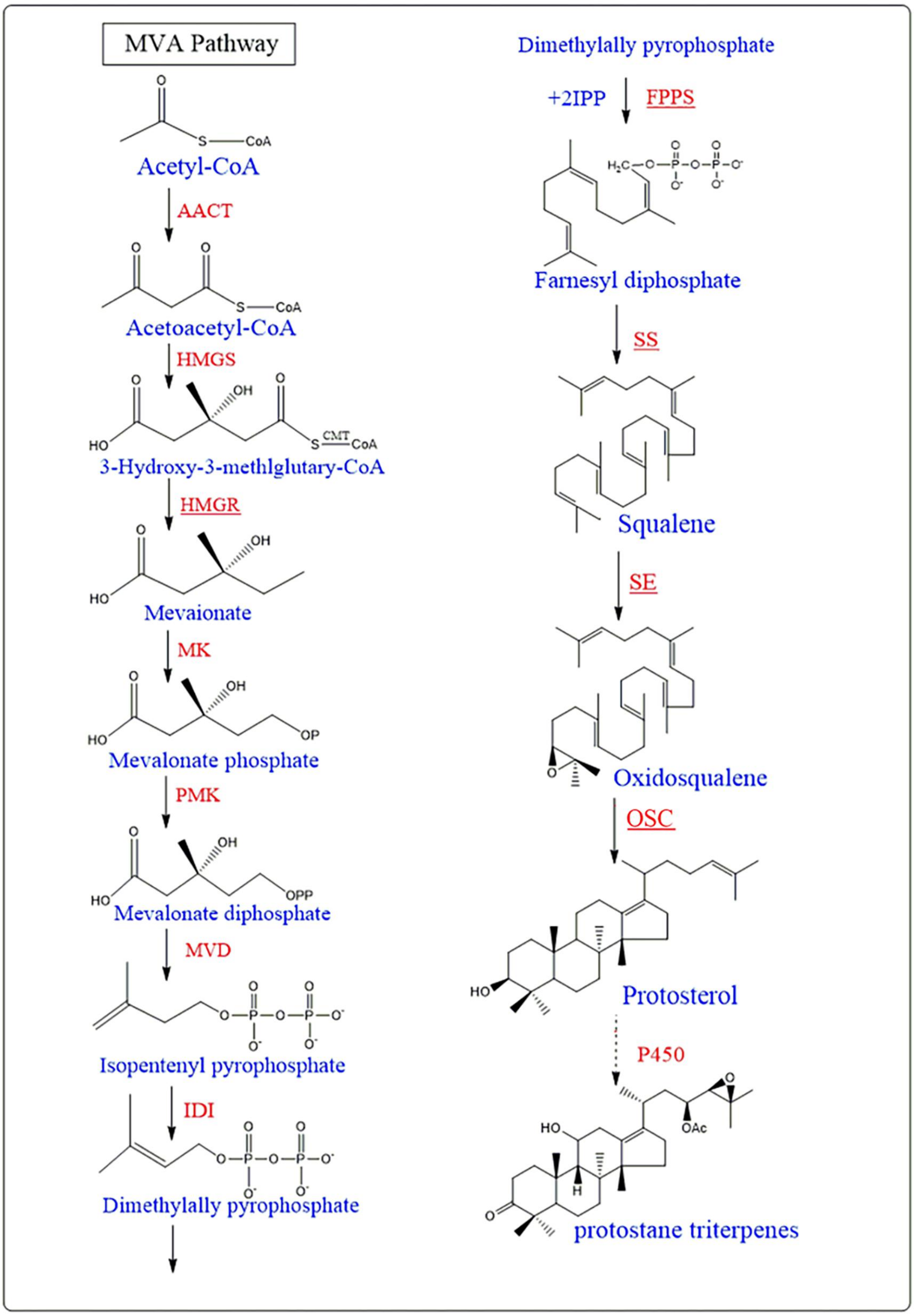

Fig. S1 The deduced biosynthetic pathway of protostane triterpene in *A. orientale.*

AACT: Acetyl-CoA acetyltrans-ferase; HMGS: 3-hydroxy-3-methylglutaryl-CoA synthase; HMGR: Hydroxy methylglutaryl-CoA reductase; MK: Meval-onate kinase; PMK: Phosphomevalonate kinase; MVD: Mevalonate-5-pyrophosphate decarboxylase; IDI: Iisopentenyl diphosphate isomerase; FPPS: Farnesyl pyrophosphate synthase; SS: Squalene synthetase; SE: Squalene epoxidase; OSC: Oxidosqualene cyclase.

Table S1 Nucleotide sequences of the primers used in this study

Primer name	Primer sequence (5′ to 3′)
AF	5′-CWYTNACHRTNGTWTGYGAYGGYTG-3′
AR	5′-GRTGDCGCATRTTRAAWGCATCHCC-3′
SF1	5′-CCAGCACCAAGCCAACAAAACAAGAGGGGATG-3′
SR1	5′- GGTAGACATCCCCTCTTGTTTTGTTGGCTTGGTGC-3′
SF2	5′-CATGATTTGGGTGGGGCAGTTGGCAGTTCTCT-3′
SR2	5′-TCTTGGCAGACCCGTCACCCATCCTGTTCTACCC-3′
SF3	5′-CTCGAGTTAGGGGATCTACCCCTGCTG-3′
SR3	5′-ACTTGAATGTCAGCAGTCAGCTGCTAGAG-3′
SF4	5′-AGACGTCCCCGGACCAATTGCACAC-3′
SR4	5′-CGGAAACAGATCCCAGTTGTACATATAGC-3′
SF5	5'-CGGAATTCATGGTGACAATCCCTTTCCT-3'
SR5	5'-ACGCGTCGACTCAATTGACTGGAGGAGC-3'
SF6	5'-CGGAATTCATGGTTGCGCTCCCGCTC-3′
SR6	5'-ACGCGTCGACTCAATTGACTGGAGGAGC-3′
UBCF	5′ -AAACGGCTACCACATCCA -3′
UBCR	5′-CACATCCCAAGGTCCAAC-3′
SF7	5′-CCTTGATTGATGATAATGGGACT-3′
SR7	5′-GATGTCTACCTTTGCGGAGC-3′
SF8	5′-CACCCATCCTGTTCTACCCA-3′
SR8	5′-GCCACTGCCGTCTTCAAATA-3′

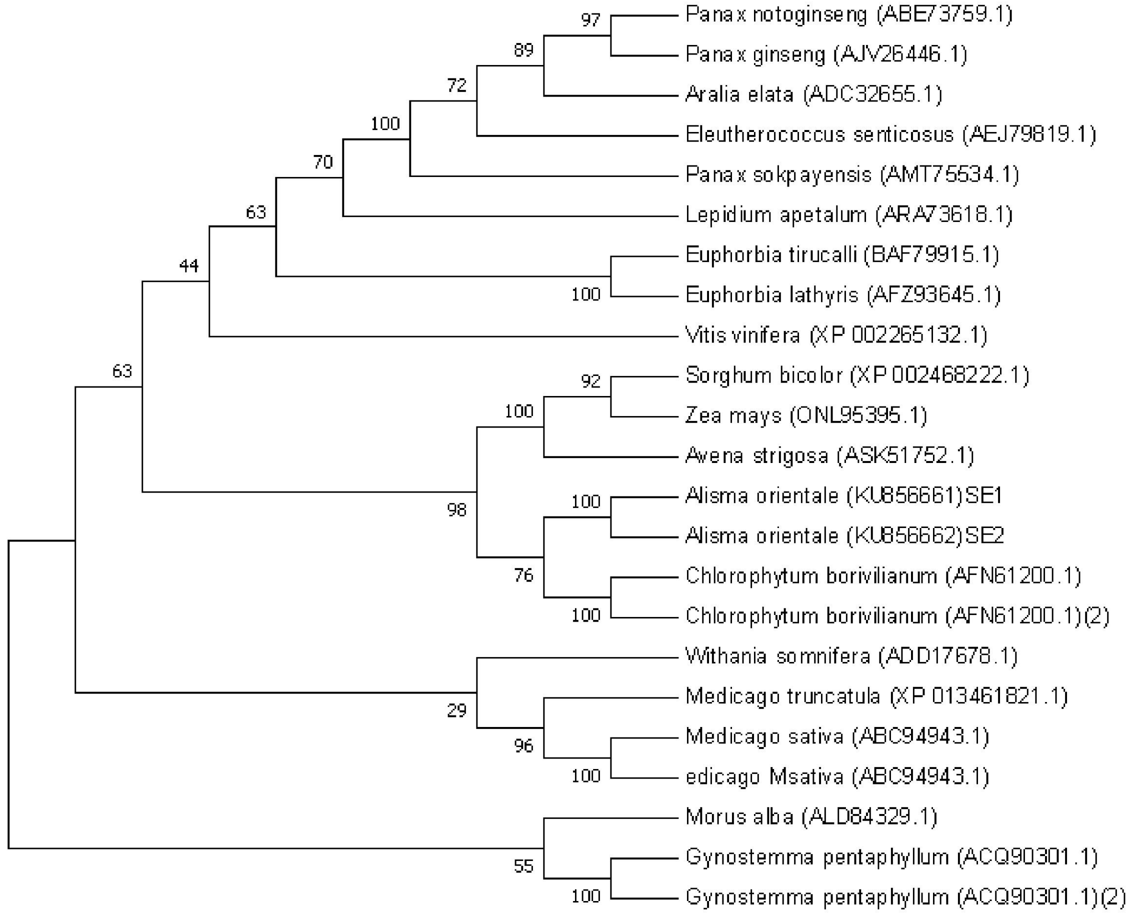

Fig. S2. Phylogenetic tree showing the relationships between the AoSE1, AoSE2 amino acid sequence and the other identified SE sequences. The tree is constructed using the neighbor-joining method in MEGA 7 and bootstrapped 1000 times. Bootstrap percentages are indicated at the branch points. In all cases, tree topologies obtained using the NJ, minimum evolution, and maximum parsimon methods are identical.

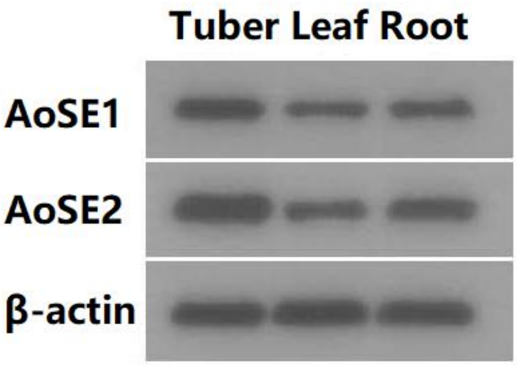

Fig. S3. Western blotting analysis of the protein AoSE1 and AoSE2 in tubers, leaves, and roots.

Table S2 Correlation analyses between AoSE1, AoSE2 expressions and alisol B 23-acetate contents

Correlation analyses	Pearson correlation
alisol B 23-acetate content and *AoSE1* mRNA expression	0.899*
alisol B 23-acetate content and *AoSE2* mRNA expression	0.958*
alisol B 23-acetate content and AoSE1 protein expression	0.913*
alisol B 23-acetate content and AoSE2 protein expression	0.954*
AoSE1 mRNA expression and AoSE1 protein expression	0.931*
AoSE2 mRNA expression and AoSE2 protein expression	0.945*

SPSS 19.0 was used for the correlation analyses between AoSEs expressions in *A. orientale* tubers and Alisol B 23-acetate contents.
* Correlation coefficient is significant at the 0.05 level (two-tailed)

Table S3 Effects of MeJA on the biomass production

Treatment	Biomass production (g)					
	0d	1d	2d	3d	4d	5d
CK	13.62±0.54	14.22±0.77	14.9±0.44	15.86±0.84	17.16±0.44	18.04±0.61
MeJA	13.72±0.53	14.28±0.50	14.92±0.54	16.04±1.31	16.88±0.93	18.28±0.28

CK: control group treated by distilled water; MeJA: sample group treated by MeJA.

Table S4 Correlation analyses between AoSE1, AoSE2 expressions and Alisol B 23-acetate contents under MeJA treatment

Correlation analyses	Pearson correlation
alisol B 23-acetate content and *AoSE1* mRNA expression	0.826*
alisol B 23-acetate content and *AoSE2* mRNA expression	0.845*
alisol B 23-acetate content and AoSE1 protein expression	0.818*
alisol B 23-acetate content and AoSE2 protein expression	0.828*
AoSE1 mRNA expression and AoSE1 protein expression	0.994**
AoSE2 mRNA expression and AoSE2 protein expression	0.989**

SPSS 19.0 was used for the correlation analyses between AoSEs expressions in *A. orientale* tubers and Alisol B 23-acetate content.

* Correlation coefficient is significant at the 0.05 level (two-tailed)

** Correlation coefficient is significant at the 0.01 level (two-tailed)
